# Estimating Sentence-like Structure in Synthetic Languages Using Information Topology

**DOI:** 10.3390/e24070859

**Published:** 2022-06-22

**Authors:** Andrew D. Back, Janet Wiles

**Affiliations:** School of Information Technology and Electrical Engineering, The University of Queensland, Brisbane, QLD 4072, Australia; j.wiles@uq.edu.au

**Keywords:** information-theoretic models, synthetic language, sentence boundary estimation, sentence-like units

## Abstract

Estimating sentence-like units and sentence boundaries in human language is an important task in the context of natural language understanding. While this topic has been considered using a range of techniques, including rule-based approaches and supervised and unsupervised algorithms, a common aspect of these methods is that they inherently rely on a priori knowledge of human language in one form or another. Recently we have been exploring synthetic languages based on the concept of modeling behaviors using emergent languages. These synthetic languages are characterized by a small alphabet and limited vocabulary and grammatical structure. A particular challenge for synthetic languages is that there is generally no a priori language model available, which limits the use of many natural language processing methods. In this paper, we are interested in exploring how it may be possible to discover natural ‘chunks’ in synthetic language sequences in terms of sentence-like units. The problem is how to do this with no linguistic or semantic language model. Our approach is to consider the problem from the perspective of information theory. We extend the basis of information geometry and propose a new concept, which we term information topology, to model the incremental flow of information in natural sequences. We introduce an information topology view of the incremental information and incremental tangent angle of the Wasserstein-1 distance of the probabilistic symbolic language input. It is not suggested as a fully viable alternative for sentence boundary detection per se but provides a new conceptual method for estimating the structure and natural limits of information flow in language sequences but without any semantic knowledge. We consider relevant existing performance metrics such as the F-measure and indicate limitations, leading to the introduction of a new information-theoretic global performance based on modeled distributions. Although the methodology is not proposed for human language sentence detection, we provide some examples using human language corpora where potentially useful results are shown. The proposed model shows potential advantages for overcoming difficulties due to the disambiguation of complex language and potential improvements for human language methods.

## 1. Introduction

In human communications, language is generally understood in chunks [1,2,3,4,5,6,7,8,9,10,11,12,13]. In spoken language, the idea of a sentence is not a straightforward notion due to the lack of textual clues, punctuation or morphological information [14]. Hence, there may be prosodic information used to determine sentence boundaries when dealing with spoken language, and the concept of sentence-like units (SLUs) is often used [15,16]. Sentences in written language are typically defined in terms of adhering to some known grammatical rules, for example, patterns of nouns, verbs, and adjectives. In the field of natural language processing, sentence segmentation is generally used as a precursor to automatic speech recognition. For convenience, we will generally use the term ‘sentence’ where the meaning of either sentence or sentence-like unit will be determined by the context.

The concept of sentences has been challenged, with some arguing that the more natural foundational unit is the phrase rather than the sentence [17]. This approach is also likely to be more consistent with topic estimation, where the aim is to discover larger phrases than precise sentence structure.

Sentence boundary detection is often widely varying in task definition [18]. Many of these models are effectively solving disambiguation tasks, where the aim is to find the most likely sentence bound from among a small number of possible tokens. More recent attention has been given to some more challenging domains, such as legal or clinical domains [19,20].

Methods for sentence segmentation typically use textual or prosodic information and sentence boundaries as input features, and then a typical approach is to train a model on corpus data to learn to predict sentence boundaries on unseen data [21]. Typically, natural language processing methods rely on learning large-scale probabilistic relationships, grammars, ontologies and functional relationships using knowledge of human languages. For example, some approaches use hidden Markov models (HMMs) [22].

A probabilistic approach for parts of speech (POS) labeling, which includes end of sentence boundaries for sentence boundary detection using conditional random fields (CRF), was proposed in [23]. In this case, a conditional probability is assigned over the label sequences given an observation sequence instead of trying to fit a joint distribution over the label and observation sequences. The CRF model can be viewed as an undirected graphical model, where random variables represent observation sequences and the nodes represent elements of the label sequence. In contrast to the HMM approach, the independence assumptions are relaxed to ensure tractable inference. Models in this category are typically parametrized using a maximum entropy algorithm requiring a large amount of labeled training data.

Some approaches to sentence segmentation have relied on rule-based models learning the difference between periods in the text as sentence boundaries and their use as other punctuation marks [24]. A model for sentence boundary detection based on a set of grammatical rules for the way in which sentences use verbs was proposed in [25]. A method for sentence boundary detection using a grammatical rule-based system to define the linking structure between words was considered in [26]. A segmentation method based on a syntactic structural model, which increased in complexity with corpus length, was proposed in [27].

A common aspect of previous models is that even though there are approaches based on rules, supervised machine learning models, or even unsupervised approaches, they inherently are derived with some knowledge of the language. This is evident in the way in which sentence boundary detection algorithms are generally evaluated by comparing the results against some known gold standard [28].

We have previously proposed a new approach to artificial intelligence based on emergent synthetic languages, which can be used to model natural behaviors using a linguistic style approach [29]. Unlike human language, however, with its infinite richness [30], synthetic language is based on the idea that the behavior of many systems may be treated within a simpler framework. In this case, probabilistically framed behavioral events derived from dynamical systems may be viewed as words within a synthetic language.

Synthetic language is based on the idea of capturing behaviors with a small alphabet, perhaps only 5–10 symbols, and a limited vocabulary. A key difference between synthetic language and human language is that there is not necessarily any teacher or knowledge of the language whatsoever in the case of synthetic language. This means that most of the techniques used in natural language processing (NLP) are of limited value for use in the proposed framework of synthetic language.

This synthetic language framework has demonstrated effectiveness on some otherwise challenging problems, for example, detecting neurological conditions by modeling conversational speech [31]. An important consideration in the development of synthetic language is the capability of determining sentence or phrase structure.

Language is generally understood to be comprised of sequences of probabilistic elements, ordered into sets of words, conforming to some grammatical rules [32]. Evidence suggests that consistent rules of grammar develop rapidly even with new languages; moreover, this is found to occur with languages other than spoken or written forms, for example, sign languages [32]. Human language has been differentiated from animal language by its use of syntactic communication, which gives rise to the combinatorial richness found in human language [33].

The probabilistic primitive elements of language are typically a small, finite set of symbols that are combined together to form words, sentences and phrases, extending to longer narratives that can be understood in terms of probabilistic principles such as Zipfian laws, which have been proposed to describe the relationship between probabilistic elements.

While the concept of emergent synthetic languages is appealing, the problem is that there is generally no initial teacher or model of the language semantics, grammar or structure. This means that recognition cannot depend on traditional approaches that assume such a priori knowledge. This is even more difficult than unsupervised learning when the languages are known and some form of background knowledge is available.

Unlike most natural language processing methods that have the advantage of a teacher with knowledge of language structure such as parts of speech, we raise the question of whether it is possible to identify synthetic language structure, such as sentences using information theoretic principles. Moreover, it is not necessarily feasible to segment such a sequence and measure the performance directly because we do not actually know where such segments should be. Therefore this raises questions of how is it possible to derive a method of segmentation in synthetic languages and how do we measure the effectiveness of a proposed model?

Our approach here is not intended to be a definitive new method for sentence boundary detection; rather, we are seeking to propose a new conceptual model for thinking about how to process completely novel languages for which there is no known teacher or background knowledge. The aim is to consider a possible way in which there may exist information-theoretic ‘sequential chunks’, which are similar to but not necessarily the same as sentences or even phrases.

Hence in this paper, we propose a new approach we refer to as *information topology,* which extends the widely known information geometry methodology [34,35]. In particular, we present a novel method that measures the incremental information flow across such a topology and show how this can be used to estimate natural bounds in sentence-like structures without any semantic knowledge.

We describe this approach in the next section, and then, in subsequent sections, explore how it can be used to effectively provide a method of discovering synthetic language structure. Given that there may be no way of measuring any actual sentence boundaries within synthetic languages, we introduce a new performance measure, which we propose will help provide a possible approach to assessing the performance of this and other algorithms similar to it in the future. We also introduce a new form of relative entropy that we term *normalized relative difference entropy,* which appears to be well suited for this particular area.

## 2. Analyzing Language Using Information Topology

### 2.1. Statistical Manifolds

Our approach in this area is to consider how a probabilistic view of synthetic language may be used to estimate structure and potentially discover the meaning of an unknown language. As noted above, the problem we face is considerably different from the usual natural language processing (NLP). The field of NLP normally relies on an a priori knowledge of any given language. In this case, however, such knowledge is not assumed to exist. In general, the one assumption we choose to invoke is that synthetic languages will have some underlying probabilistic structure in common with human languages.

This means that we might expect to see that there exists a Zipfian structure across different levels of language. In addition, we expect that the language will consist of a small set of primitives that are random but occur with some probabilistic consistency. Such symbols might then be grouped to form synthetic words and sentences. A synthetic language might also be considered in terms of parts of speech, grammar, lexicons and other familiar aspects of language; however, this does not seem to be a strict requirement in the same way as encountered in human language. For example, it is not clear how parts of speech as a language construct may be instantiated as the alphabet size and vocabulary size change.

The main aspect of this probabilistic approach to determining language meaning is that we are interested in methods of discovering language structure based only on probabilistic measurements. In previous work, we have proposed a number of algorithms that can be useful for determining synthetic language symbols and words. The next level we propose to consider is segmenting sentences (or some approximation of them) from a sequence of synthetic language words.

The approach we propose to consider is if the information flow can be used to segment sequences into sentence-like units. Moreover, we are interested in determining if the structural aspects of information flow are related to the structural aspects of language sequences. Hence, we firstly consider the information geometry approach as a way of understanding this information flow.

A convenient starting point in our discussion is to consider the concept of relative information-theoretic measurements. The information-theoretic properties of a natural sequence can be defined in terms of the self-information

(1) $$H_{0}\left(X\right)=E\left[I(s)\right]$$

where the expectation can be defined in terms of the probabilities of each element

(2) $$I\left(s\right)=\log_{2}\left(p(s)\right)$$

Now, this results in the single symbol Shannon entropy defined as [36]:

(3) $$H_{0}\left(X\right)=-\sum_{i=1}^{M}p\left(x_{i}\right)\log_{2}\left(p\left(x_{i}\right)\right)$$

Entropy can be considered to describe the level of ‘surprise’ or information content in a given sequence of probabilistic data and extends to the case where the probabilities of multiple symbols occurring together are taken into account. Entropy-based measures have been applied to a range of tasks, including the use of decision trees for character recognition [37], analysis of physiological patterns for emotion detection [38], cluster analysis [39], face recognition [40], identification of disease markers through human gene mapping [41,42] and detection of covert communications by analyzing the patterns of packet timing events [43]. While entropy is useful for characterizing the probabilistic nature of language, a problem exists with trying to estimate relatively rare events from limited data [44].

It can be observed that there is an inherent distance between statistical elements. A convenient model for determining the distance between distributions is relative entropy (also known as the Kullback–Leibler divergence [35,45]), which is defined as

(4) $$H_{R}\left(X;Y\right)=\frac{1}{2}\sum_{i=1}^{M}p\left(x_{i}\right)\log_{2}\left(\frac{p\left(x_{i}\right)}{p\left(y_{i}\right)}\right)$$

While relative entropy is useful for contrasting pairs of distributions, this raises the question of how to contrast a sequence of distribution pairs. When comparing multiple sequences of data with different distributions, the  Kullback–Leibler divergence or relative entropy is typically used. However, there are various ways in which differences can exist. For example, consider a simple probability mass function, then, a single point can account for most of the divergence, or it may be due to a small change across the entire function. The implications of each may be quite different, and hence, this means we may need to consider this concept of contrasting relative distributions in more detail.

**Figure 1:**
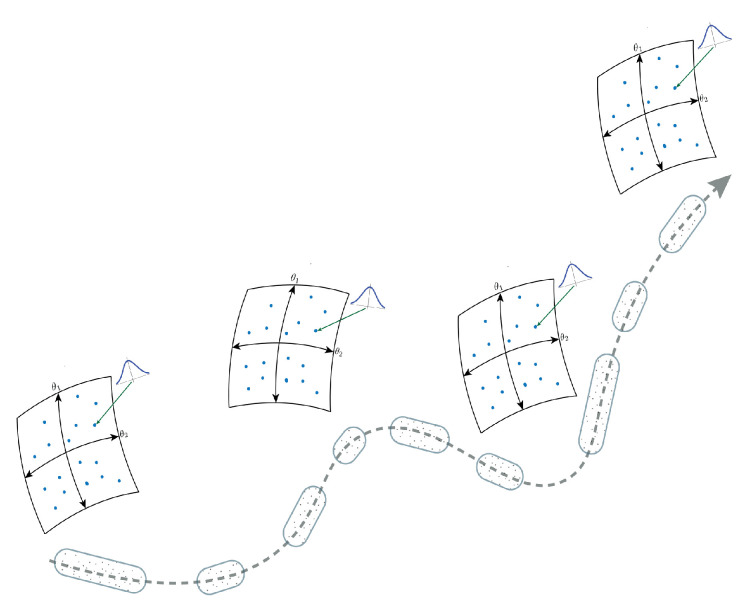
Information flow can be considered in terms of the incremental changes in relative distributions over time. Here, we visualize the concept of multiple curved Riemannian manifold spaces formed in a language sequence. Can this probabilistic structure be used to reveal some aspects of the language sequence structure?

Another way in which we can consider the issue of probabilistic divergences is through the concept of information geometry, where each distribution can be considered to exist as a point in a statistical manifold, and such manifolds are not necessarily flat as in a usual Euclidean space but may be curved. Moreover, within the context of language, we are not interested in simple differences between two points (i.e., two distributions) only but between the broader differences between sequences of distributions. This means we need a way to measure these differences and understand what such differences mean. A visual representation of this idea is shown in Figure 1. We give more explicit detail to this below by considering the concepts of statistical manifolds and information geometry in relation to multiple probability distributions.

An information-theoretic approach to analyzing language can be formulated on the basis of understanding the relationships that may exist between different distributions. Consider a family of probability distributions $S=\left\{p(x,\theta )\right\}$, which may be termed a statistical model over some space *X* with observable random variable x∈X, where each distribution p(x,θ) is parametrized by an n-dimensional real vector, forming a coordinate system $\theta =[\theta_{1},\ldots ,\theta_{n}].$ Hence, *S* can be regarded as an n-dimensional statistical manifold where each point in the space, labeled by coordinates θ represents a probability distribution. In this case, *S* is a Riemannian manifold, where the distance between two distributions can be measured by the Kullback–Leibler divergence. The classical information-geometric formulation is based on the idea of examining the local properties of curves and surfaces in the statistical space [46,47,48].

This approach provides a foundation for understanding the relationships that may exist between different distributions. We consider an extension to this idea in terms of continuously changing distributions over time, which gives rise to the concept of information topology. In the subsequent sections below, we discuss an approach for measuring the information topology space, particularly in regard to probabilistic symbolic sequences of language.

### 2.2. Contrasting Distributions on a Riemannian Manifold

A question of significant practical interest is how to detect statistical anomalies observed in natural systems such as behavioral dynamics [31] through consideration of distributions on a statistical manifold. In this case, it is necessary to compare natural symbolic sequences in terms of their probabilistic behavior.

The conventional approach to measuring the distance between distributions on a Riemannian manifold can be achieved by relative entropy [36,49]. In this approach, we view natural language as discrete random variables *X* of a sequence *X* = $X_{1},\ldots ,X_{i},\ldots ,X_{K}$ $,X_{i}=x\in \;$X${}^{M},$ that is, $x_{i}$ may take on one of *M* distinct values, $X^{M}$ is a set from which the members of the sequence are drawn, and hence, $x_{i}$ is in this sense symbolic, where each value occurs with probability $p\left(x_{i}\right),$ i∈[1,M].

Suppose we wish to contrast two sequences of social behavioral data; this might occur within various contexts such as conversational dialog, swarms, geopolitical events or human–machine interaction. Now, instead of contrasting direct time-series data, our interest is in information-theoretic modeling. Is it possible to derive an understanding of the underlying system by considering the changes in the relative distributions over time?

**Figure 2:**
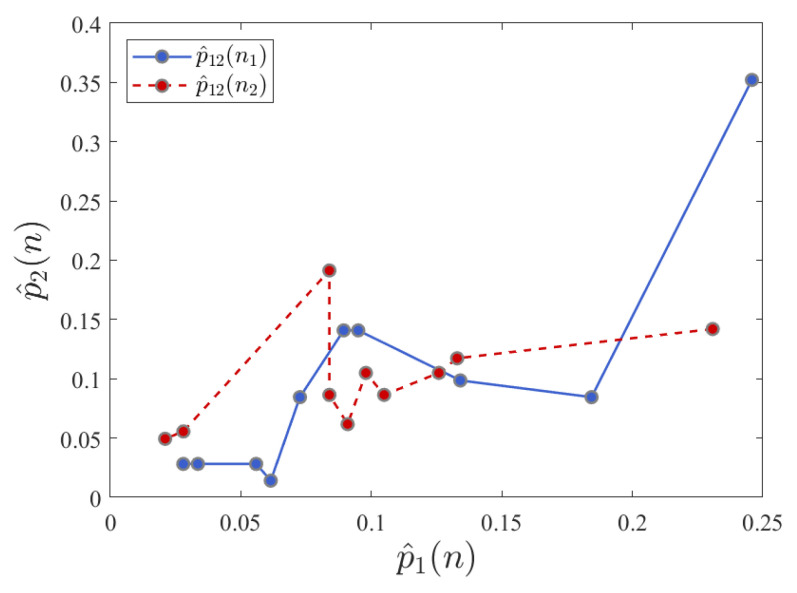
A view of contrasting pairs of probability distribution trajectories from a natural sequence. Each curve represents points on distributions of successive points plotted against each other. Hence, changes in the underlying probability characteristics can be visualized across the sequence and can be considered in terms of traversing a Riemannian manifold. Can this view of information flow be used to analyze structure in synthetic language sequences?

As an example of this, consider the changing probability distributions in a synthetic language sequence. In this case, audio conversation files are transformed into synthetic languages based on an alphabet size of 10 symbols, using the pause lengths between speech audio activity [31,50]. A visualization of the trajectories of changes in probability distributions computed from sequences of natural conversational data is shown in Figure 2. In this particular case, we consider only two of the probability mass points, $\left\{{\hat{p}}_{12}\left(n_{a}\right),{\hat{p}}_{12}\left(n_{b}\right)\right\},$ where ${\hat{p}}_{12}\left(n_{a}\right)$ indicates the probability located at the trajectory point $\left[{\hat{p}}_{1}\left(n_{a}\right),{\hat{p}}_{2}\left(n_{a}\right)\right]$ at time $n_{a}.$ Hence, this enables the comparison of corresponding points in probability trajectory space over time. Our task is then to determine a more comprehensive probabilistic model of the underlying behavior that gives rise to the observed probabilistic changes.

In the next section, we develop an approach to addressing this issue using a model based on statistical curvature from information geometry, extended to consider the shape of information flow.

### 2.3. Normalized Ollivier–Ricci Curvature

The approach we are interested in is to consider the notion of statistical curvature as a method of understanding the structure and potentially some aspects of meaning within synthetic language. The idea is that it is not only the distance between probability distributions that may be useful but the curvature and potentially the shape of the manifold.

As an introduction to the ideas contained in this section, consider the idea of relative differences between distributions. Suppose we have one distribution, which for convenience, we consider in terms of a point mass function. A starting point to measure the difference between this distribution and another is to measure the relative entropy. This essentially provides a direct measurement of the difference. However, as noted in [31], a degeneracy exists, which means that there is an essentially infinite number of distributions that can exist that have the same relative entropy values. Therefore, what are we to do?

A further method of contrasting distributions is to measure the transport distance. This is also known as the earth-mover distance and intuitively provides an indication of the nearness between points in the two distributions required to make one the same as the other.

Now, based on these two distance measures, it is possible to introduce the concept of a type curvature in Riemannian probability space. Consider two separate examples of pairs of distributions. For a constant relative entropy in each pair, it is possible to formulate a measure that indicates the change between the two pairs based on the transport distance. Hence, if  one of these pair measurements is greater than the other, we might say that it is because the manifold is more curved. This is the idea behind Ricci curvature and then made explicit in the Ollivier–Ricci curvature [51].

More formally, the statistical curvature between distributions in Riemannian space can be extended to a sectional curvature model on a Riemannian manifold. Given a Riemannian manifold (X,d), (*X* is a metric measure space equipped with distance *d*), consider two tangent vectors $\left\{v,w_{x}\right\}$ at a point x∈X, then parallel transport the unit vector $w_{x}$ from *x* to *y*, which is the end-point of δv where ε,δ>0. The sectional curvature K(v,w) at *x* is defined over all directions *w* where [51]

(5) $$d=\delta \left(1-\frac{\varepsilon^{2}}{2}K\left(v,w\right)+O\left(\varepsilon^{3}+\varepsilon^{2}\delta \right)\right)$$

A simplified formulation is the Ricci curvature, which averages K(v,w) over all directions w. The Ollivier–Ricci curvature is a coarse approximation to the Ricci curvature given by

(6) $$\kappa \left(x,y\right)=1-\frac{W_{1}\left(u_{x},u_{y}\right)}{d(x,y)}$$

where $\left\{u_{x}:x\in X\right\}$ is a family of probability measures on the manifold and $W_{1}\left(u_{x},u_{y}\right)$ is the Wasserstein-1 transportation distance given by

(7) $$W_{1}\left(u_{x},u_{y}\right)={\left(\underset{\xi \in \Pi (u_{x},u_{y})}{\inf}\;\int \int \;d{\left(x,y\right)}^{p}du\left(x,y\right)\right)}^{1/p}$$

where $\Pi (u_{x},u_{y})$ is a set of all couplings between measures $u_{x}$ and $u_{y}.$ The transportation distance from $u_{x}$ to $u_{y}$ represents the shape of the curve or the effective distance between the spheres $u_{x}$ and $u_{y}$, and d(x,y) is the distance between the centers of $u_{x}$ and $u_{y}.$ The distance d(x,y) is the minimum path between vertices on a graph or ‘hop’ distance. While the direct minimum path between vertices is appropriate for network graphs, relative entropy or Kullback–Leibler divergence provides a measure of the distance between the elements of ranked order probability distributions.

Ricci curvature has found application in numerous areas to characterize high dimensional complex probabilistic data, including internet topology [52], cancer studies [53] and phylogenetics [54]. Hence, as a means of applying a probabilistic curvature model to synthetic language, we introduce a normalized Ollivier–Ricci curvature measure defined as

(8) $$\tilde{\kappa }\left(x,y\right)=1-\frac{{\tilde{W}}_{1}\left(u_{x},u_{y}\right)}{{\tilde{H}}_{R}\left(u_{x},u_{y}\right)}$$

where ${\tilde{H}}_{R}(u_{x},u_{y};$x) is the normalized relative entropy across $x=\left\{x,y\right\}$ given by

(9) $${\tilde{H}}_{R}\left(u_{x},u_{y}\right)=\frac{1}{\left(1-\pi_{L}\right)}\left(\frac{H_{R}\left(u_{x},u_{y}\right)}{\pi_{H}}-\pi_{L}\right)$$

and

(10) $$H_{R}\left(x,y;M\right)=-\sum_{i=1}^{M}p_{i}\left(x\right)\log_{2}\left(\frac{p_{i}\left(x\right)}{p_{i}\left(y\right)}\right)$$

is the usual relative entropy measure with scaling factors $\left\{\pi_{L},\pi_{H}\right\}$ given by

(11) $$\begin{array}{c} \pi_{L}=\underset{n}{\inf}\left\{H_{R}\left(n\right),n\in \left[1,N_{a}\right]\right\} \end{array}$$

(12) $$\begin{array}{c} \pi_{H}=\underset{n}{\sup}\left\{H_{R}\left(n\right),n\in \left[1,N_{a}\right]\right\} \end{array}$$

where $N_{a}$ is the sequence length, expressed in terms of the number of segments from which the relative entropy measure is computed, with index n. Similarly, the normalized Wasserstein-1 transportation distance ${\tilde{W}}_{1}\left(u_{x},u_{y}\right)$ is given by

(13) $${\tilde{W}}_{1}\left(u_{x},u_{y}\right)=\frac{1}{\left(1-\xi_{L}\right)}\left(\frac{W_{1}\left(u_{x},u_{y}\right)}{\xi_{H}}-\xi_{L}\right)$$

with scaling factors $\left\{\xi_{L},\xi_{H}\right\}$ given by

(14) $$\begin{array}{c} \xi_{L}=\underset{n}{\inf}\left\{W_{1}\left(n\right),n\in \left[1,N_{a}\right]\right\} \end{array}$$

(15) $$\begin{array}{c} \xi_{H}=\underset{n}{\sup}\left\{W_{1}\left(n\right),n\in \left[1,N_{a}\right]\right\} \end{array}$$

A limitation of this method is that it implicitly assumes the cardinality of $\left\{u_{x},u_{y}\right\}$ is identical for each distribution. However, this assumption is typically not valid in practice, and so a method is required to overcome this issue. Hence, we introduce the normalized relative difference entropy, which solves the problem and is defined as follows.

Suppose we have measures $\left\{u_{v},u_{z}\right\}$ where $p_{v}=[p_{1}\left(v\right),\ldots ,$$p_{n_{v}}\left(v\right)]$ and $p_{z}=[p_{1}\left(z\right),$$\ldots ,p_{n_{z}}\left(z\right)]$ are the distributions associated with $\left\{u_{v}\right\}$ and $\left\{u_{z}\right\}$ of dimension $n_{v}$ and $n_{z}$, respectively, where $n_{v}\neq n_{z}.$ We introduce associated measures $\left\{u_{\hat{v}},u_{\hat{z}}\right\}$ where $p_{\hat{v}}=\left[p_{1}\left(\hat{v}\right),\ldots ,p_{n_{\hat{v}}}\left(\hat{v}\right)\right]$ and $p_{\hat{z}}=\left[p_{1}\left(\hat{z}\right),\ldots ,p_{n_{\hat{z}}}\left(\hat{z}\right)\right]$ are the distributions associated with $\left\{u_{\hat{v}}\right\}$ and $\left\{u_{\hat{z}}\right\}$ of dimension $n_{\hat{v}}$ and $n_{\hat{z}}$, respectively, where $n_{\hat{v}}=n_{\hat{z}}.$ The associated distributions are found as

(16) $$\begin{array}{c} p_{\hat{v}}=f_{s}\left(p_{v};\theta_{\hat{v}}\right) \end{array}$$

(17) $$\begin{array}{c} p_{\hat{z}}=f_{s}\left(p_{z};\theta_{\hat{z}}\right) \end{array}$$

where $f_{s}$ is an interpolated spline function parametrized by $\left\{\theta_{\hat{v}},\theta_{\hat{z}}\right\},$ resulting in matched distributions $\left\{p_{\hat{v}},p_{\hat{z}}\right\}$, which can be then applied to determine the matched relative entropy as

(18) $$H_{m}\left(v,z;n_{\hat{v}}\right)=-\sum_{i=1}^{n_{\hat{v}}}p_{i}\left(\hat{v}\right)\log_{2}\left(\frac{p_{i}\left(\hat{v}\right)}{p_{i}\left(\hat{z}\right)}\right)$$

with scaling to obtain ${\tilde{H}}_{m}\left(v,z;n_{\hat{v}}\right)$ according to Equations (11) and (12) as before. Similarly, the matched normalized Wasserstein-1 transportation distance ${\tilde{W}}_{1m}\left(u_{\hat{v}},u_{\hat{z}}\right)$ is given in the same way, with scaling according to Equations (13) and (14).

We can now apply this normalized Ollivier–Ricci curvature to synthetic language sequences of symbolic data. This provides an indication of the change in the probabilistic structure of the language being used over time. For the proposed information topology approach, to achieve an effective measure of the changes in information, we define a new form of relative entropy called the normalized relative difference entropy. This is defined as

(19) $$H_{D}\left(\hat{v},\hat{z};n_{\hat{v}}\right)=-\frac{1}{n_{\hat{v}}}\sum_{i=1}^{n_{\hat{v}}}\left(\frac{{\left(p_{i}\left(\hat{v}\right)-p_{i}\left(\hat{z}\right)\right)}^{2}}{p_{i}{\left(\hat{z}\right)}^{2}}\right)\log_{2}\left(\frac{p_{i}\left(\hat{v}\right)}{p_{i}\left(\hat{z}\right)}\right)$$

Adopting the same scaling principle as indicated in Equations (9)–(12), leads to the scaled version of the normalized relative difference entropy given by

(20) $${\tilde{H}}_{D}\left(u_{\hat{v}},u_{\hat{z}}\right)=\frac{1}{\left(1-\pi_{L}\right)}\left(\frac{H_{D}\left(u_{\hat{v}},u_{\hat{z}}\right)}{\pi_{H}}-\pi_{L}\right)$$

Hence, we can introduce a normalized difference Ollivier–Ricci curvature measure defined on matched distributions $\left\{p_{\hat{v}},p_{\hat{z}}\right\}$ as

(21) $${\tilde{\kappa }}_{D}\left(\hat{v},\hat{z}\right)=1-\frac{{\tilde{W}}_{1m}\left(u_{\hat{v}},u_{\hat{z}}\right)}{{\tilde{H}}_{D}\left(u_{\hat{v}},u_{\hat{z}}\right)}$$

The curvature of the Riemannian manifold that supports the family of probability distributions indicates an information geometry. However, in terms of recognition of the flow of dialog in synthetic language, our interest is in forming a global view of the probabilistic nature of language with local features. Can this be extended further to estimate synthetic language structure? In the next section, we extend the normalized Ollivier–Ricci curvature to an information topology space.

### 2.4. Information Topology Manifold

To introduce a topology into the Riemannian manifold, one approach is to note that ${\tilde{W}}_{1m}\left(u_{\hat{v}},u_{\hat{z}}\right)$ defines an arc in the space, which is subtended by the distance ${\tilde{H}}_{D}\left(u_{\hat{v}},u_{\hat{z}}\right).$ Hence, the chord distance is related to the radius $r_{\hat{v}\hat{z}}$ by the function

(22) $$r_{\hat{v}\hat{z}}=f_{r}\left({\tilde{H}}_{D}{\tilde{W}}_{1};u_{\hat{v}},u_{\hat{z}}\right)$$

where we omit the *m*-subscript for notational convenience, with the matched probabilistic inputs indicated by context, and  $r_{\hat{v}\hat{z}}$ is found by solving the function $f_{r}$ according to

(23) $${\tilde{H}}_{D}\left(u_{\hat{v}},u_{\hat{z}}\right)=2r_{\hat{v}\hat{z}}\sin\left(\frac{{\tilde{W}}_{1}\left(u_{\hat{v}},u_{\hat{z}}\right)}{2r_{\hat{v}\hat{z}}}\right)$$

and where ${\tilde{H}}_{D}\left(u_{\hat{v}},u_{\hat{z}}\right)$ and ${\tilde{W}}_{1}\left(u_{\hat{v}},u_{\hat{z}}\right)$ are determined as above. Now, this indicates a particular sectional arc angle, which can readily be found as

(24) $$\theta_{\hat{v}\hat{z}}=\frac{{\tilde{W}}_{1}\left(u_{\hat{v}},u_{\hat{z}}\right)}{r_{\hat{v}\hat{z}}}$$

We extend the notion of a curved probabilistic manifold across multiple points (each representative of a distribution in the information space). Hence, for each pair of points $\left(u_{\hat{v}},u_{\hat{z}}\right)$ a related arc angle will be obtained.

For any two points, it is possible to derive a circle, and for multiple points, a hypersphere of appropriate dimensionality can be obtained defined by the set $\left\{\theta_{\hat{v}\hat{z}}\right\}$, thereby defining the required topological features on the manifold. Note that Equation (23) is generally well behaved, and hence, $\theta_{\hat{v}\hat{z}}$ can be easily obtained by numerical solution.

The normalized Ollivier–Ricci sectional radius derived from the curvature of natural sequence symbolic data can be extended to the sectional arc angle. An information topology space can be obtained by extending the concept of curvature to a higher dimensional manifold.

One approach to achieve this is by extending the normalized Ollivier–Ricci sectional arc angle to an n-dimensional sphere in n-dimensional parameter space. This effectively transforms a sequential symbolic set into an event-based representation. Note that a sequence of symbols may be sampled in the time-domain or indexed from some other feature space. Extending this to the sectional arc angle, the normalized Ollivier–Ricci sectional arc angle can be derived from the curvature of natural sequence symbolic data and applied to a set of synthetic language data.

The concept of an information topology extends information geometry to create a new approach to viewing information. This extends the idea of a symbolic entropy-based event space beyond the natural curvature measures to one in which we might possibly consider higher-dimensional shapes and topology. Our idea is that in contrast to simpler classification approaches, this potentially gives a framework for probabilistic metalanguages to be mapped into these spaces and to represent intrinsic meaning using the lexical components of the synthetic language via mappings and topological features on a Riemannian manifold and the grammatical components through the dynamic patterns in this space.

Once we have obtained the sectional arc angles, it is straight-forward to generate a representation of this using multidimensional hyperspheres, where the information topology manifold can be formed across any number of parameter dimensions. The parameters can be derived using various probabilistic estimation algorithms; see, for example, [55].

In contrast to conventional Euclidean manifolds, where the distance between points is measured by simple straight lines (i.e., using a Pythagorean metric), here, information topology manifolds provide a new approach for potentially understanding the meaning of sequences of synthetic language. This extends the concept of measuring information content in data by enabling the distances between probability distributions to be measured using entropy-based divergence metrics to capture the information properties in a manifold.

The advantage of this approach is that almost any natural sequence that can be symbolized and subsequently described in terms of a synthetic natural language with dynamic probabilistic distributions can be modeled in terms of an information topology. Using this approach, it is possible to consider multidimensional measures of synthetic language in a higher dimensional information topology. This framework indicates the possibility of associating meaning to natural sequences through feature recognition on an information topology manifold.

This approach to deriving an information topology is considered and more explicitly implemented in the next section, where sequences of hyper-dimensional distribution segments form contrasting topological regions that yield insights into the unfolding structure of language sequences.

## 3. Information-Theoretic Sentences

### 3.1. Incremental Relative Information

The segmentation of sentences or phrases in synthetic language is made difficult by the potential lack of knowledge of the language itself. Hence, this means that conventional approaches to determining sentences based on language aspects such as symbols, parts of speech, grammar, words or punctuation are not likely to be feasible due to the lack of such properties. An alternative approach is, therefore, to consider some form of probabilistic approach using minimal assumptions about the language.

Earlier approaches that adopt this idea of complex language structure identification combining probabilistic information with language instantiation are considered in various contexts. A model mapping words, linguistic and contextual factors to a prosodic probabilistic information structure was proposed in [56]. A review of sophisticated probabilistic models of language processing and acquisition was given in [57].

A probabilistic model of learning developed within a Bayesian framework showed that surprise signals modulate learning speed, hence giving insight into constraints on statistical theories of animal and human learning [58]. It was shown in [59] that humans attempt to learn confidence-weighted transition probabilities underlying auditory and visual sequences.

In the previous section, we considered an approach to modeling the information topology of a natural sequence with a view of observing the probabilistic characteristics of the sequence. The idea of this is that the probabilistic properties mimic the structure of the sequence in terms of the information being conveyed. This raises the question of whether it may be possible to model the information flow in finer detail and, hence, derive an information-theoretic model to detect sentence or phrase boundaries.

A simple approach is the concept that for each sentence, we expect that there will be a limit to how much information is conveyed. This could potentially provide an information bound and hence determine when the sentence ends. However, the problem with this approach is that sentences can carry varying amounts of information, and hence, there is a need for further probabilistic constraints to determine sentence or phrase bounds. Note that information content is not necessarily dependent on sentence length. A long rambling sentence may contain little new information, but a short sentence might have surprising content.

The approach we propose can be considered unsupervised since it does not employ a language model or a set of labeled training data. However, it is substantially different from other unsupervised methods. For example, a sentence segmentation algorithm proposed by Kiss and Strunk is regarded as unsupervised since it does not employ a language model; however, it does rely on inherent knowledge of the language features, such as knowing what constitutes a period and the potential end of sentence [60].

In contrast, our proposed method does not use any labeled training data, language model or grammatical features or knowledge of the parts of speech. Hence, our proposed approach can be referred to as a blind model [61] since it is based on fundamental mathematical properties without regard to general linguistic properties.

A further information-theoretic constraint can be considered in terms of not only the absolute value of information carried but a more subtle measure of the completeness of information conveyed. For example, it may be possible to measure the change or even deceleration of information conveyed or even characterized in terms of the shape of the information flow over the course of a sentence. Hence, we propose a model for estimating sentence boundaries based on measuring the probabilistic characteristics of incremental information change.

The next aspect to consider is the particular language elements to use as a basis for measuring information. In practice, there are numerous possible choices. For the purpose of our investigation, we propose that a suitable proxy of information change is the n-grams of symbolic elements. The incremental information is defined by measuring the change in information due to a new set of n-grams, which have not been observed in the previous sequence.

This differs from other computational linguistic approaches, for example, where we seek to form large-scale predictive probabilistic models using all possible words. The difficulties of this are evident both in terms of data requirements, sparseness of examples, computational and linguistic complexity. We proceed to explore this approach as follows.

Suppose that we have a sequence of n-grams given by $S\left(n_{g}\right)=\left[s_{0},\ldots ,s_{N}\right],$ where we explicitly specify the size of the n-grams as $n_{g}$ in length, each of which is treated as a symbol and may take any value out of a prescribed set of available n-grams but may be comprised of individual language elements. The use of unique symbols enables the basis for precisely measuring new incremental information. Then, the Shannon information is defined in a similar manner to Shannon entropy, based on the probabilities of the observed elements. Note that the probabilities can be measured in terms of the known long-term probabilities or in terms of some shorter length history.

The Shannon self-information due to the occurrence of a single probabilistic symbolic event is defined according to Equation (2). We can generalize this to permit the occurrence of an event at a particular time n, where the probability is a function of both the particular symbol and the time (or context) in which it may occur. In this case, we have:

(25) $$I_{k}\left(s;n\right)=-\log_{2}\left(p(s_{k};n)\right)$$

The average self-information across all events with associated probabilities defines the entropy. Suppose there exists a sequence of independent symbolic events observed at time n, given by $\psi_{n},$ then the total of all self-information is given by

(26) $$I_{0}\left(\psi ;n\right)=\sum_{i\in \psi_{n}}I_{i}\left(s\right)$$

and for a sequence $\psi_{n-1},$ it follows that

(27) $$I_{0}\left(\psi ;n-1\right)=\sum_{k\in \psi_{n-1}}I_{k}\left(s\right)$$

Now, consider the set of unique incremental symbols is defined as

(28) $$\phi_{n}=\psi_{n}-\sum_{j=1}^{N_{L}}\psi_{n-j}$$

where $N_{L}$ is the immediate, short-term context length for which we consider the relevance of past information in terms of a potential sentence-like unit, and hence, we can define the incremental information $I_{d}\left(\phi ;n\right)$ at time n, as

(29) $$I_{d}\left(\phi ;n,N_{L}\right)=I_{0}\left(\psi ;n\right)-I_{0}\left(\bar{\psi }\left(N_{L}\right);n-1\right)$$

and where

(30) $${\bar{\psi }}_{n-1}\left(N_{l}\right)=\sum_{j=1}^{N_{L}}\psi_{n-j}$$

Typically the incremental information is found using a block-wise overlap-add method, which provides a convenient approach to measuring the information gained over small steps in the sequence by giving a contrastive measure to the previous short history. The  cumulative incremental information that occurs as a result of the incremental set of symbols $\phi_{n}$ is defined as

(31) $$\hat{I}\left(\phi ;n\right)=\sum_{n\in \pi }I_{d_{}}\left(\phi ;n,N_{L}\right)$$

where π is the set of all segments in a sequence of language elements.

In a similar way, the incremental normalized Wasserstein-1 distance ${\tilde{W}}_{d1}\left(\phi ;n,N_{L}\right)$ can be determined operating on the incremental or newly added unique set of symbols $\phi_{n}$ to a sequence, that is, the difference between the current set of observed language elements $\psi_{n}$ and the recent contextual set of language elements ${\bar{\psi }}_{n-1}\left(N_{l}\right).$ Another view of this is that it is the incremental distance to the next novel set of n-grams $\phi_{i+1}\left(t\right)$ not observed in the recent sequence $\{\phi_{i\in t}\left(t\right)\}.$ Hence, the incremental normalized Wasserstein-1 distance is given by

(32) $${\tilde{W}}_{d1}\left(\phi ;n,N_{L}\right)={\tilde{W}}_{1}\left(\psi ;n,N_{L}\right)-{\tilde{W}}_{1}\left(\bar{\psi };n-1,N_{L}\right)$$

where ${\tilde{W}}_{1}\left(\phi ;n,M\right)$ is computed according to Equations (13) and (14). Hence, the cumulative incremental Wasserstein-1 distance, which occurs as a result of the incremental set of symbols $\phi_{n}$, is defined as

(33) $${\hat{W}}_{1}\left(\phi ;n,N_{L}\right)=\sum_{n\in \pi }{\tilde{W}}_{d1}\left(\phi ;n,N_{L}\right)$$

The idea of this approach is that we are concerned with determining the incremental flow of information with symbols in a sequence and how this might be used to gain insight into the potential meaning of the sequence. In particular, we are interested in the question of whether the change in probabilistic information might provide some means of determining the natural bounds on a chunk of a sequence.

In our subsequent derivation, we adopt the broad assumption of a Zipfian probabilistic structure of language primitives and propose that these can be estimated using a previously derived model, which requires a small number of data points [44]. An  important aspect of this process is the question of how natural language sequences are converted into synthetic language symbols. This is addressed in our previous work, where a number of symbolization algorithms have been derived [29]. In addition, a key aspect of the proposed model is that the probabilities of short segments of symbolic sequences can be reliably estimated with limited data. This is achieved by means of a previously proposed algorithm described in detail in [55].

### 3.2. Curvature of Incremental Tangent Normalized Wasserstein Distance

The incremental information gain considered in the previous section provides an effective starting point to determine sentence boundaries by measuring the cumulative information over a sequence of language elements. Now, in addition to a language sequence information flow, we might expect that there will be some form of “connectedness”, where the sequential information elements are probabilistically related and, in an information topology sense, converges over the course of a sentence. The idea is that we can measure the packaging of information within a sentence and, hence, require a measure of the convergence of the information flow in some sense. 

Here, we propose to consider the Wasserstein distance in conjunction with relative entropy to measure the curvature of the information space. We estimate the incremental normalized Wasserstein distance ${\tilde{W}}_{d1}\left(\phi ;n\right)$ between short segments of language elements, where, as indicated previously and in the example shown here, we use novel n-grams $\phi_{i+1}\left(t\right)$ not observed in the sentence $\{\phi_{i\in t}\left(t\right)\}.$

**Figure 3:**
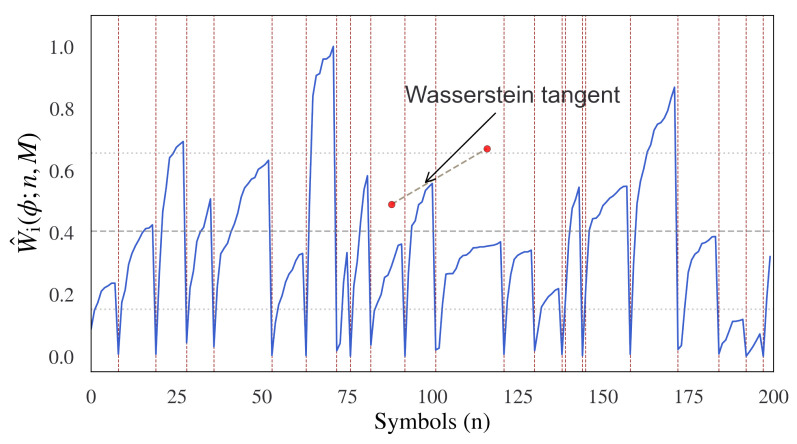
The cumulative incremental Wasserstein distance for n-grams is shown here for a range of sentences in the Brown News corpus. Here, each sentence is marked by the vertical red lines. It can be observed that the curvature increases rapidly for each sentence to a limit before each new sentence begins. The curvature of the Wasserstein distance increases rapidly for each sentence and then tapers off. This can be understood in terms of the tangent angle of the Wasserstein distance, which measures the decreasing change in incremental information as each sentence progresses. The x axis is shown in terms of information-carrying symbols, and the y-axis is in terms of cumulative incremental Wasserstein distance.

The decreasing curvature of the information flow can be estimated using the cumulative incremental normalized Wasserstein-1 distance and adopted as a measure of the sentence boundaries. This can be visualized across sequential segments, and for demonstrative purposes, an example of this measure applied to the Brown News corpus [62] is shown in Figure 3. 

Interestingly, as can be observed in the example, the curvature of the Wasserstein distance decreases as the sentence progresses. This can be viewed in terms of the tangent angle of the Wasserstein distance, which measures the decreasing change in incremental information along the sequence. While the absolute value of the information flow in terms of the curvature of the Wasserstein distance may vary significantly for each sentence, and the change in curvature is remarkably consistent. This provides a potential basis for confirming the initial idea that the information change will decrease as each sentence progresses and, hence, permit the possible identification of sentence boundaries. 

The proposed algorithm does not require any language model or other form of labeled training data. Apart from the assumption of Zipfian structure, we do not introduce any form of a priori grammatical structure or insight into the language properties. In this sense, as noted above, the algorithm can be considered a blind, unsupervised approach.

We note that there are limitations with this approach, indicating the requirement for further investigation. In particular, the method is based on the notion of incremental information changes, as measured by the curvature of the Wasserstein distance. However, it is evident that for some of the phrases tested, there can be difficulties in accurately measuring this. While the measure is generally accurate, it is possible to find cases where the information flow is not so consistent. For example, if a speaker trails off in their voice, does this indicate the end of a sentence or not? Hence, it might be of interest to introduce further prosodic or other multidimensional symbolization approaches that could enhance the model estimation process [63]. 

A normalization factor is applied and the curvature of the tangent is found as

(34) $${\tilde{\theta }}_{1}\left(\phi ;n\right)=\arctan\left({\hat{W}}_{1}\left(\phi ;n\right)\right)$$

The decreasing curvature of the information flow, as estimated using the cumulative incremental normalized Wasserstein-1 distance, provides insight into the shape of the information flow. One approach to estimate the sentence boundaries is to model the curvature at the end of each sentence and then estimate the sentence boundary based on this curvature directly. For  example, in the simplest case, a maximum likelihood estimator could be used to determine a limit on the curvature, which would provide a test to determine the sentence bound.

A more sophisticated approach is to use a combined EM-HMM approach, where an EM algorithm is used to estimate a set of curvature bounds [64], and an HMM model [65] is used to estimate which state we are in based on the observed sequence of information changes as measured in terms of either the incremental information or the incremental normalized Wasserstein-1 distance. The aim here is to determine that, according to a particular input sequence, detecting a particular curvature can then indicate the end of a sentence. 

It is also evident that there are various other observation sequences, such as prosodic, morphological or semantic sequences, which can be used to train an HMM model to select the end of a sentence. These methods are beyond the scope of this paper, and thus, we do not consider them further here. Rather, we present a simple decision region approach that can be used to identify the end of sentences. 

The idea behind our approach is simply that there is a limited amount of information carried by a sentence or sentence-like unit. Unlike approaches that carry some outside form of sentence boundary indicator, whether textual or prosodic, our approach is based entirely on information theoretic methods. The basis of our method is that by introducing a proxy of information, we can measure the decreased flow of information as each sentence progresses and, hence, based on the historical context of decision bounds, make a reasonable estimate of when the sentence is ending. The decision bounds used to indicate when this information flow can be determined theoretically or estimated approximately using a training algorithm on contextual data. 

In particular, our approach here is to recognize that it is possible to estimate the sentence boundaries by combining the cumulative information $\hat{I}\left(\phi ;n\right)$ and the decreasing curvature of the cumulative incremental normalized Wasserstein-1 distance ${\tilde{\theta }}_{1}\left(\phi ;n\right).$ Hence, a bounded region $\Omega (\phi_{N})$, which will be used for a decision-making process, can be determined based on these parameters. The idea here is that the information topological parameters $\left\{{\tilde{\theta }}_{1}\left(\phi ;n\right),\hat{I}\left(\phi ;n\right)\right\}$ can be tracked throughout a dialog and then used to initiate an impending end of sentence, and then when the boundary of the decision region is reached, an end of sentence is flagged. The bounded region is defined as

(35) $$\Omega \left(\phi_{N}\right)=\left[\omega_{l},\omega_{b},\omega_{w},\omega_{h}\right]$$

where *N* is the number of symbols used for the model, and $\left[\omega_{l},\omega_{b},\omega_{w},\omega_{h},\omega_{t}\right]$ defines a region consisting of left, bottom, width and height parameters, respectively, given by

(36) $$\begin{array}{c} \omega_{l}=\bar{I}\left(\phi ;n\right)-\alpha \psi_{w} \end{array}$$

(37) $$\begin{array}{c} \omega_{b}=\bar{\theta }\left(\phi ;n\right)-\alpha \psi_{h} \end{array}$$

(38) $$\begin{array}{c} \omega_{w}=\alpha \psi_{w} \end{array}$$

(39) $$\begin{array}{c} \omega_{h}=\alpha \psi_{h} \end{array}$$

where $\omega_{t}=\omega_{b}+\omega_{h}$ and a covariance matrix of the cumulative information $\hat{I}\left(\phi ;n\right)$ and cumulative incremental normalized Wasserstein-1 distance ${\tilde{\theta }}_{1}\left(\phi_{n};n,M\right)$ is given by

(40) $$\Sigma_{I\theta }=\Sigma \left(\bar{I}\left(\phi ;n\right),\bar{\theta }\left(\phi ;n\right)\right)$$

with eigenvalues $\left(\lambda_{1},\lambda_{2}\right)$ and eigenvector $v_{1}$ and where $\bar{I}\left(\phi ;n\right)$ and $\bar{\theta }\left(\phi ;n\right)$ are the means of the maximum values of $\hat{I}\left(\phi ;n\right)$ and ${\tilde{\theta }}_{1}\left(\phi_{n};n\right)$ computed as

(41) $$\bar{I}\left(\phi ;n\right)=\frac{1}{N_{p}}\sum_{n=1}^{N_{p}}\max\left(\hat{I}\left(\phi ;n\right)\right)$$

and

(42) $$\bar{\theta }\left(\phi ;n\right)=\frac{1}{N_{p}}\sum_{n=1}^{N_{p}}\max\left(\tilde{\theta }\left(\phi ;n\right)\right)$$

where $N_{p}$ is considered the long-term historical context and defines the number of sentence-like units in the proceeding history. This defines a covariance ellipse as

(43) $$\Psi \left(\phi_{N}\right)=\left[\psi_{w},\psi_{h},\psi_{c},\psi_{a}\right]$$

where $\psi_{w},\psi_{h},\psi_{c},\psi_{a}$ are parameters of width, height, center and angle, respectively, and α is a scaling parameter (α=0.5 for unity standard deviation region) and  

(44) $$\begin{array}{c} \psi_{w}=2\lambda_{1} \end{array}$$

(45) $$\begin{array}{c} \psi_{h}=2\lambda_{2} \end{array}$$

(46) $$\begin{array}{c} \psi_{c}=\left(\bar{I}\left(\phi ;n\right),\bar{\theta }\left(\phi ;n\right)\right) \end{array}$$

(47) $$\begin{array}{c} \psi_{a}=\arccos\left(v_{1}\right) \end{array}$$

**Figure 4:**
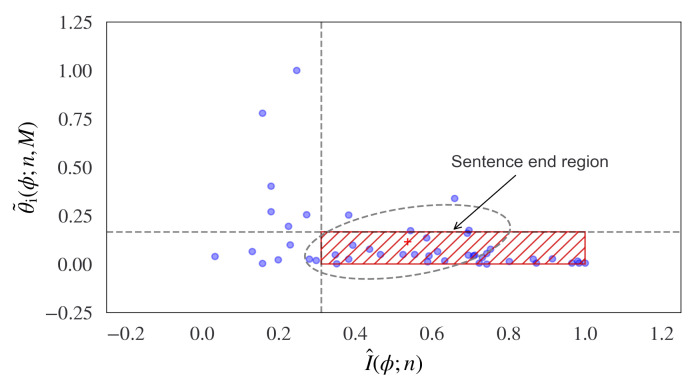
A method of analyzing structure in synthetic language is shown using information topology. In this measure, the elliptical region indicates the end of a sentence. This is found as the constrained limit between the information flow and the decreasing change in curvature of the information flow. This is given by the probabilistic curvature measurements of the cumulative incremental tangent angle of the estimated Wasserstein-1 distance (y axis) and the cumulative incremental information (x axis). The results are shown for a range of known sentences in the Brown News corpus. The red hatched region defines the bound of the information flow and predicts the sentence end-points.

Hence, a bounded region can be determined, which enables sentences to be determined in synthetic language sequences. Examples of this bounded region for a range of known sentences in the Brown News corpus are displayed in Figure 4, where the red hatched area indicates the bounded information flow region, which provides a method of estimating synthetic language sentence boundaries.

Once the trajectory of a sentence crosses into this region, it indicates the sentence ending. This decision point q$\left(\phi ;n\right)$ for an end of a sentence-like unit is indicated as

(48) $$\;q\left(\phi ;n\right)=\left\{\begin{array}{cc} 1 & \mathrm{if}\;\left(\hat{I}\left(\phi ;n\right)\geq \omega_{l}\right)\&\left(\tilde{\theta }\left(\phi ;n\right)\leq \omega_{t}\right)\\ 0 & \mathrm{otherwise} \end{array}\right.$$

**Figure 5:**
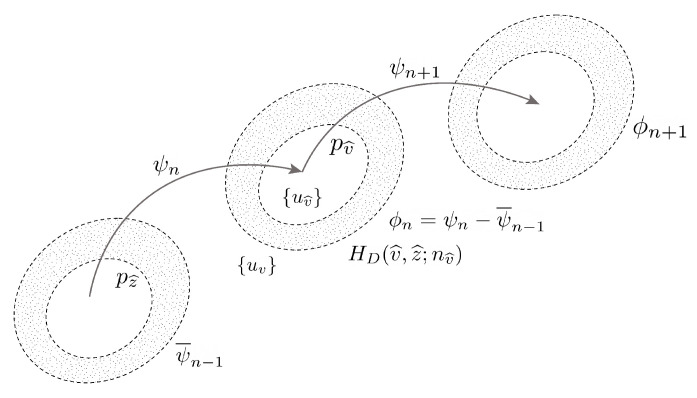
A diagrammatic representation of the information topological algorithm measuring the probabilistic curvature measurements between short segments of symbolic sequences. The curvature diminishes to a bound on the information flow, predicting the sentence end-point.

The pseudo-code for the proposed algorithm is shown in Algorithm 1, where an initial procedure computes the decision bounds and then is used by a second procedure on current input data, and a visual representation of the algorithm is shown in Figure 5. 

**Figure 6:**
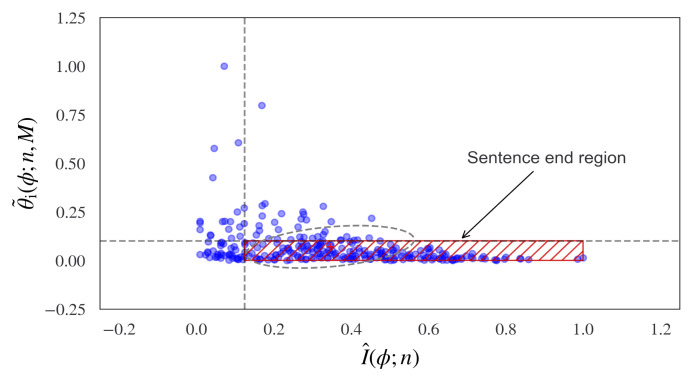
The probabilistic curvature measurements of the cumulative incremental tangent angle Wasserstein-1 distance (y axis) and the cumulative incremental information (x axis) are shown for 200 known sentences in the Brown News corpus. The clustering shows evidence of the expected information change for each sentence. The red hatched region defines the bounds of the information flow and predicts the sentence end-points.

The experimental results for a range of known sentences in the Brown News corpus are shown in Figure 6, where a learning region can be determined, which can be used to indicate the end of a sentence.

**Figure 7:**
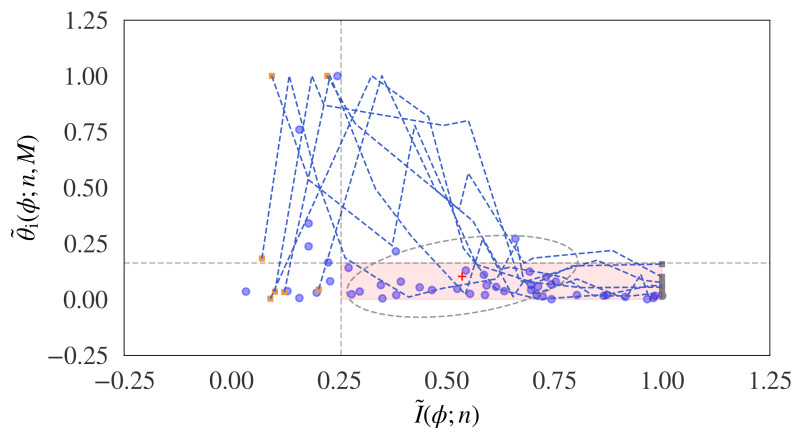
The trajectories of the probabilistic curvature measurements of the cumulative incremental tangent angle Wasserstein distance (y axis) and the cumulative incremental information (x axis) are shown for 10 known sentences in the Brown News corpus. The sentence end-points are detected when the trajectory crosses into the red hatched region. The results indicate the potential of the approach for determining the sentence bounds.

The trajectories of the probabilistic curvature measurements of the cumulative incremental tangent angle Wasserstein distance and the cumulative incremental information are shown for 10 known sentences in the Brown News corpus in Figure 7.
**Algorithm 1** Proposed information topology SLU estimation algorithm1:**procedure**InfTopSentenceBounds($\Pi_{h}$)         ▹ Estimate contextual SLU bounds2:    **for all** $g\in \Pi_{h}$ **do**                   ▹ Do for all contextual data $\Pi_{h}$3:        $\left\{u_{v},u_{z}\right\}\leftarrow g\left(\Pi_{h}\right)$                   ▹ Read new set of symbols4:        $\left\{u_{\hat{v}},u_{\hat{z}}\right\}\leftarrow \left\{u_{v},u_{z}\right\}$       ▹ Obtain topological set of new unique symbols5:        $\left\{p_{\hat{v}},p_{\hat{z}}\right\}\leftarrow f_{s}\left(\left\{u_{v},u_{z}\right\}\right)$         ▹ Functional spline match distributions6:        $H_{D}\left(\hat{v},\hat{z}\right)\leftarrow \left\{p\left(\hat{v}\right),p\left(\hat{z}\right)\right\}$       ▹ Normalized relative difference entropy7:        $I_{d}\left(\phi \right)\leftarrow I_{0}\left(\psi \right)-I_{0}\left(\bar{\psi }\left(N_{L}\right)\right)$            ▹ Incremental information8:        $\hat{I}\left(\phi ;n\right)\leftarrow I_{d}$               ▹ Cumulative incremental information9:        ${\tilde{W}}_{d1}\left(\phi ;n\right)={\tilde{W}}_{1}\left(\psi ;n\right)-{\tilde{W}}_{1}\left(\bar{\psi };n-1\right)$  ▹ Incr. norm. Wasserstein-1 distance10:        ${\hat{W}}_{1}\left(\phi ;n\right)\leftarrow {\tilde{W}}_{d1}\left(\phi ;n\right)$              ▹ Cumulative W-1 distance11:        ${\tilde{\theta }}_{1}\left(\phi ;n\right)=\arctan\left({\hat{W}}_{1}\left(\phi ;n\right)\right)$         ▹ Curvature of the W-1 distance tangent12:    **end for**13:    $\bar{I}\left(\phi ;n\right)\leftarrow mean\left(\hat{I}\left(\phi ;n\right)\right)$           ▹ Mean cumulative information14:    $\bar{\theta }\left(\phi ;n\right)\leftarrow mean\left({\tilde{\theta }}_{1}\left(\phi_{n};n\right)\right)$         ▹ Mean incremental W-1 distance15:    $\Psi \left(\phi_{N}\right)\leftarrow \Sigma \left(\bar{I}\left(\phi ;n\right),\bar{\theta }\left(\phi ;n\right)\right)$    ▹ Covariance of incr. info and W-1 distance16:    $\omega_{l}=\bar{I}\left(\phi ;n\right)-\alpha \psi_{w}$17:    $\omega_{b}=\bar{\theta }\left(\phi ;n\right)-\alpha \psi_{h}$18:    $\omega_{w}=\alpha \psi_{w}$19:    $\omega_{h}=\alpha \psi_{h}$20:    $\Omega \left(\phi_{N}\right)=\left[\omega_{l},\omega_{b},\omega_{w},\omega_{h}\right]$          ▹ Compute decision bounds over $\Pi_{h}$21:**end procedure**22:**procedure**InfTopSentence($G_{s},\Omega_{\phi }$)          ▹ Estimate SLU for current data23:    **for all** $g\in G_{s}$ **do**                    ▹ Do for all local data $G_{s}$24:        $\left\{u_{v},u_{z}\right\}\leftarrow g\left(G_{s}\right)$                  ▹ Read new set of symbols25:        $\left\{u_{\hat{v}},u_{\hat{z}}\right\}\leftarrow \left\{u_{v},u_{z}\right\}$       ▹ Obtain topological set of new unique symbols26:        $\left\{p_{\hat{v}},p_{\hat{z}}\right\}\leftarrow f_{s}\left(\left\{u_{v},u_{z}\right\}\right)$        ▹ Functional spline match distributions27:        $H_{D}\left(\hat{v},\hat{z}\right)\leftarrow \left\{p\left(\hat{v}\right),p\left(\hat{z}\right)\right\}$           ▹ Norm. relative difference entropy28:        $I_{d}\left(\phi \right)\leftarrow I_{0}\left(\psi \right)-I_{0}\left(\bar{\psi }\left(N_{L}\right)\right)$             ▹ Incremental information29:        $\hat{I}\left(\phi ;n\right)\leftarrow I_{d}$                ▹ Cumulative incremental information30:        ${\tilde{W}}_{d1}\left(\phi ;n\right)={\tilde{W}}_{1}\left(\psi ;n\right)-{\tilde{W}}_{1}\left(\bar{\psi };n-1\right)$       ▹ Incr. norm. W-1 distance31:        ${\hat{W}}_{1}\left(\phi ;n\right)\leftarrow {\tilde{W}}_{d1}\left(\phi ;n\right)$                ▹ Cumulative W-1 distance32:        ${\tilde{\theta }}_{1}\left(\phi ;n\right)=\arctan\left({\hat{W}}_{1}\left(\phi ;n\right)\right)$        ▹ Curvature of W-1 distance tangent33:        $\Omega \left(\phi_{N}\right)=\left[\omega_{l},\omega_{b},\omega_{w},\omega_{h}\right]$             ▹ Apply SLU decision bounds34:        **if** $\left(\hat{I}\left(\phi ;n\right)\geq \omega_{l}\right)\;\mathrm{and}\;\left(\tilde{\theta }\left(\phi ;n\right)\leq \omega_{t}\right)$ **then**         ▹ SLU decision test35:           $q\left(\phi ;n\right)=1$                      ▹ End of SLU detected36:        **end if**37:    **end for**38:**end procedure**

### 3.3. F-Measure Performance Analysis

Measuring the performance of linguistic processing algorithms is a non-trivial process due to the different metrics that may be considered and the way in which they are weighted. In particular, the concept of precision and recall have been used to measure errors associated with substitution, deletion and insertion [66]. 

At the lowest level, performance can be assessed by comparing the result of matching a tag in a reference set that represents ground truth against a hypothesis [67]. At each tag location, there may be one or more slots, for example, the tags in the context of detecting sentence boundaries might consist of slots corresponding to a period, question mark or exclamation mark [28]. Hence, the precision and recall measures are defined as:

(49) $$P=\frac{C}{C+\hat{M}},\;R=\frac{C}{C+\hat{N}}$$

where *P* is the precision measure determined as the number of correct results *C* divided by the number of actual outcomes, that is, the total number of slots in the hypothesis, and *R* is the recall measure calculated as the number of correct results divided by the number of possible outcomes, that is, the total number of slots in the reference. The number of correct results is considered those where the slots in the hypothesis are exactly aligned with slots in the reference. The various slot errors can be used to determine the total number of actual and possible error outcomes as

(50) $$\begin{array}{c} \hat{M}=S+I \end{array}$$

(51) $$\begin{array}{c} \hat{N}=S+D \end{array}$$

where *S* is the number of errors due to the wrong slot being substituted, *D* is the number of errors due to a slot being in the reference set being missed in the hypothesis set, and *I* is the number of errors due to a slot being flagged in the hypothesis that does not exist in the reference set. The F-measure can be defined as the harmonic mean of the precision and recall values [68]

(52) $$F_{\alpha }=\frac{RP}{\left(1-\alpha \right)P+\alpha R}\;\mathrm{for}\;\;0\leq \alpha \leq 1$$

A common expression is

(53) $$F=\frac{2RP}{R+P}$$

where α=0.5. The F-measure has been criticized since it implicitly over-emphasizes some particular types of errors compared to others [67], which could lead to bias [69]. In particular, risk-centric applications may give a high weight to retrieving information more so than precision, whereas a high F-measure can occur due to an uneven weighting between precision and recall; hence, variations based on different scaling values α have been proposed such as the F2-measure [70] and the semantic error rate [71].

A problem exists in devising an appropriate performance measure for synthetic language because, although we can conduct a test on known human languages, this does not necessarily inform us about the actual expected performance in a synthetic language environment.

A particular issue is that we do not necessarily have any access to the true end-of-sentence boundaries, so it is not possible to apply existing performance measures. Nevertheless, as a preliminary test of the performance of the proposed algorithm, we conducted a test on a known human language corpus and compared it against an existing sentence boundary detection algorithm.

We selected the Brown News corpora and compared the performance against the Kiss and Strunk (KS) algorithm [60]. We note that this is not strictly a fair comparison since our proposed method does not rely on any semantic knowledge but only uses the information topology approach. This places it at a considerable disadvantage to other algorithms, such as the KS method, which incorporates an inherent knowledge of human language. Hence, it is not expected that the BW algorithm is likely to perform as well as methods that have this advantage.

A further difference between the algorithms considered here is that the KS method is essentially a disambiguation approach as it seeks to determine the precise ending symbol. However, in our proposed algorithm, since we do not incorporate any semantic knowledge, there is no particular consideration given to any symbol as a sentence boundary. While this could be incorporated into future versions of this method, for the present version, we do not use this approach. Instead, due to the nature of our proposed algorithm, there is a margin allowed in the precise sentence ending permitted.

The results for both the KS method and the proposed BW method when applied to sentence boundary detection on the difficult text of the Brown News corpus are shown in Table 1. Interestingly, when we examine the specific sentence boundaries selected, it is evident that the KS method has difficulties in the very problem of disambiguating the use of periods within the complex text. However, the BW method does not have this same problem, which indicates that there may be a possibility of deriving a more accurate model for human language sentence boundary detection by incorporating our proposed methodology into existing algorithms such as the KS method.

### 3.4. An Information-Theoretic Performance Measure

Although we have demonstrated the performance in terms of the F-measure, in general, for synthetic language algorithms, it is not possible to know if the algorithm is operating successfully because there is not necessarily any language knowledge to indicate the ground truth. Hence, existing performance measures such as accuracy and the F-measure will not be suitable or possible to use in synthetic language data. In this section we propose such a global performance measure based on information-theoretic principles. Hence, it can be used to determine the overall effectiveness of algorithms for estimating sentence boundaries even without semantic knowledge.

We propose a global performance criterion to measure the effectiveness of the proposed approach by considering the distribution of the resulting sentence lengths. Unlike coarse methods, which might seek to determine an average sentence length and arbitrarily limit each sentence length, the method proposed here does not introduce any specific sentence limits.

Hence, the distribution of sentence lengths resulting from the proposed information topology sentence bound model can be compared against an estimated probabilistic synthetic language model.

For natural sequences, including natural language, a mechanism to model the symbolic probabilities is to use a Zipfian law [72,73]. Our approach is to use a previously derived analytic model. Hence, for a natural sequence with alphabet size M, which consists of symbols with rank r, the probability of occurrence of a given word can be defined in terms of rank, the Zipf–Mandelbrot–Li law provides an expression for the probability to be used, where [44,74,75]:

(54) $$\hat{P}\left(r;\hat{M}\right)=\frac{\gamma^{\prime }}{{\left(r+\beta \right)}^{\alpha }}$$

and for iid samples, the constants can be computed as [72]:

(55) $$\alpha =\frac{\log_{2}\left(\hat{M}+1\right)}{\log_{2}\left(\hat{M}\right)},\;\beta =\frac{\hat{M}}{\hat{M}+1},\;\gamma_{M}=\frac{{\hat{M}}^{\alpha -1}}{{\left(\hat{M}-1\right)}^{\alpha }}$$

and $\gamma^{\prime }=\gamma /\kappa$ where

(56) $$\sum_{i=1}^{M}p\left(i\right)=1,\;\sum_{i=1}^{M}\frac{\gamma }{{\left(r+\beta \right)}^{\alpha }}=\kappa$$

Having then estimated ${\hat{P}}_{h}\left(r,M\right),$ the entropy can then be easily estimated as

(57) $${\hat{H}}_{1}\left(r,X\right)=-\sum_{h=1}^{\hat{M}}{\hat{P}}_{h}\left(r,M\right)\log_{2}\left({\hat{P}}_{h}\left(r,M\right)\right)$$

which defines the rank *r* Shannon entropy estimate. However, in our case, we use the analytic distribution above to contrast against the sentence distribution obtained as a result of the incremental information topology algorithm for sentence estimation described in the previous section.

**Figure 8:**
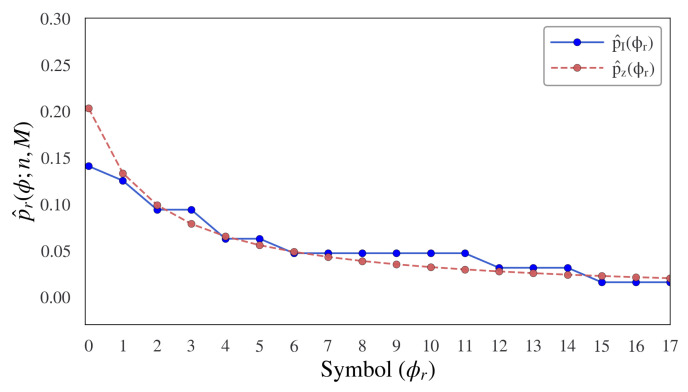
A performance criteria to measure the effectiveness of the proposed information topological sentence model is obtained by comparing the probability distributions of sentence lengths resulting from the proposed information topology sentence bound model when compared against an estimated probabilistic synthetic language model based on a Zipf–Mandelbrot–Li distribution on sentence length [44]. The distribution of the estimated model provides a reasonably similar distribution to the actual data obtained from the Brown News corpora (1000 sentence result shown).

Accordingly, we evaluate the proposed information topology approach by applying the probabilistic distribution criteria to a set of data from the Brown corpora and then comparing it to an analytic distribution as indicated here. The results for 1000 estimated sentences using this approach are shown in Figure 8. Hence, a trivial next step is to introduce a relative entropy measure to contrast the measured and expected distributions.

An interesting further example of the performance can be obtained by comparing the actual sentence data from test corpora and the estimated sentences. We suggest that a global performance metric as defined above is likely to provide a better overall indicator; however, we include it here out of curiosity. Note that all punctuation is removed for the model, an example of this is shown below:

Actual sentence: *“Only a relative handful of such reports was received”, the jury said, “considering the widespread interest in the election, the number of voters and the size of this city”*.

Estimated sentence: *“Only a relative handful of such reports was received”, the jury said, “considering the widespread interest in the election, the number of voters and the size of this city”. The jur*

Using this approach, it would be possible to formulate a metric that measures the precise effectiveness of the model against a test set. This can be achieved in a number of ways, for example, using a simple sentence length measure, the probabilistic distributions shown above, a relative entropy measure, or some other approach such as using the specific roles of words included or not. However, this example indicates that the potential of the proposed information topological method for sentence estimation in synthetic languages has been demonstrated in this section.

## 4. Conclusions

Determining sentence-like units and sentence boundaries for human language has been considered using various approaches in the literature. In this present work, we are interested in seeking to determine a method for estimating sentence-like units in synthetic language, for which there is no teacher, no grammar and very limited functional knowledge. In previous methods for human languages, even when using unsupervised methods, there is inherently some background knowledge of the language, which is normally included.

The challenge for synthetic languages is that, in contrast to human languages, we do not necessarily have any a priori knowledge of language elements. It is evident that without such a comprehensive background knowledge of a given language in terms of understanding of what constitutes language primitives such as letters, words, sentences, topics and even spaces or pauses, for synthetic languages, even determining how to identify these elements is non-trivial.

In traditional approaches, the idea of chunking sequences implies a precise aspect of determining sentence structure. However, since this is not possible, we propose a new information theoretic approach to identify synthetic language structure when there is practically nothing known about the language. The assumption we make in our development is that the language follows a Zipfian structure.

While various information-theoretic approaches, for example, based on entropy, may be useful, in this paper, we consider an extension to the information-geometric framework. In particular, we consider the notion of information topology based on the curvature in a statistical manifold unfolding over time as the sequence of language progresses. This gives the potential for synthetic language structure to be efficiently inferred through measurements in a topological information space.

To determine sentence-like structure in synthetic languages, we have proposed a model based on the concept that sentences are constrained to convey a finite amount of information in a particular shape that can be measured. We describe this approach and then show how it can be used to model the property of an information shape across a sequence based on a measure of the cumulative incremental tangent angle Wasserstein-1 distance. This curvature of this information surface is used to estimate a natural limit of information flow in language sequences. This provides the potential for a method of autonomous segmentation without any semantic or other linguistic knowledge.

Measuring the performance of the proposed algorithm is an important task, and so we have considered this in the paper. We have provided an example comparison of the proposed method by comparing it to an existing algorithm (Kiss and Strunk (2006)) and evaluated it using the F-measure. We describe the limitations of the comparison and the appropriateness of the F-measure, in general, for this work. It is evident that existing performance metrics such as the F-measure are unsuitable for synthetic language applications when there may be no language model present that can be used to indicate ground truth. Accordingly, we have introduced a novel information-theoretic that is capable of measuring the global performance based on modeled distributions.

We demonstrate the proposed sentence-like boundary estimation method and global performance measure by applying them to a human language corpora, where it is shown that the proposed approach is capable of segmenting synthetic language data into sentences that approximate known sentence structure. However, we stress that this is a new conceptual approach, and there is much work to be conducted to improve the performance of the method so that it can be effectively used.

An area of further investigation is that while the method proposed here does not consider semantic information, meaning there is no inherent grounding to re-align sentence-like units, the proposed approach has the potential to be useful in developing new methods in modeling dialog for which there is very little known about the language. In particular, given the problems that existing algorithms can have with the disambiguation of complex language, there appears to be an interesting way forward to develop more robust sentence segmentation models that overcome these problems by introducing the proposed information topology methods.

## Figures and Tables

**Table 1:** F-measure result on the Brown News corpus.

Model	$F_{\alpha }\left(\%\right)$
KS	78.91
BW	68.09

## Data Availability

The Brown corpus data are available as part of the NLTK from https://www.nltk.org/book/ch02.html (accessed on 3 August 2021).
